# Supplementary material for: A comprehensive list of the Bunyavirales replication promoters reveals a unique promoter structure in Nairoviridae differing from other virus families
Source: Sci Rep. 2022 Aug 9;12:13560. doi: 10.1038/s41598-022-17758-z (PMC9363447; doi:10.1038/s41598-022-17758-z)
Supplement: Supplementary file 4 — Supplementary Figures. [file 41598_2022_17758_MOESM4_ESM.pdf]

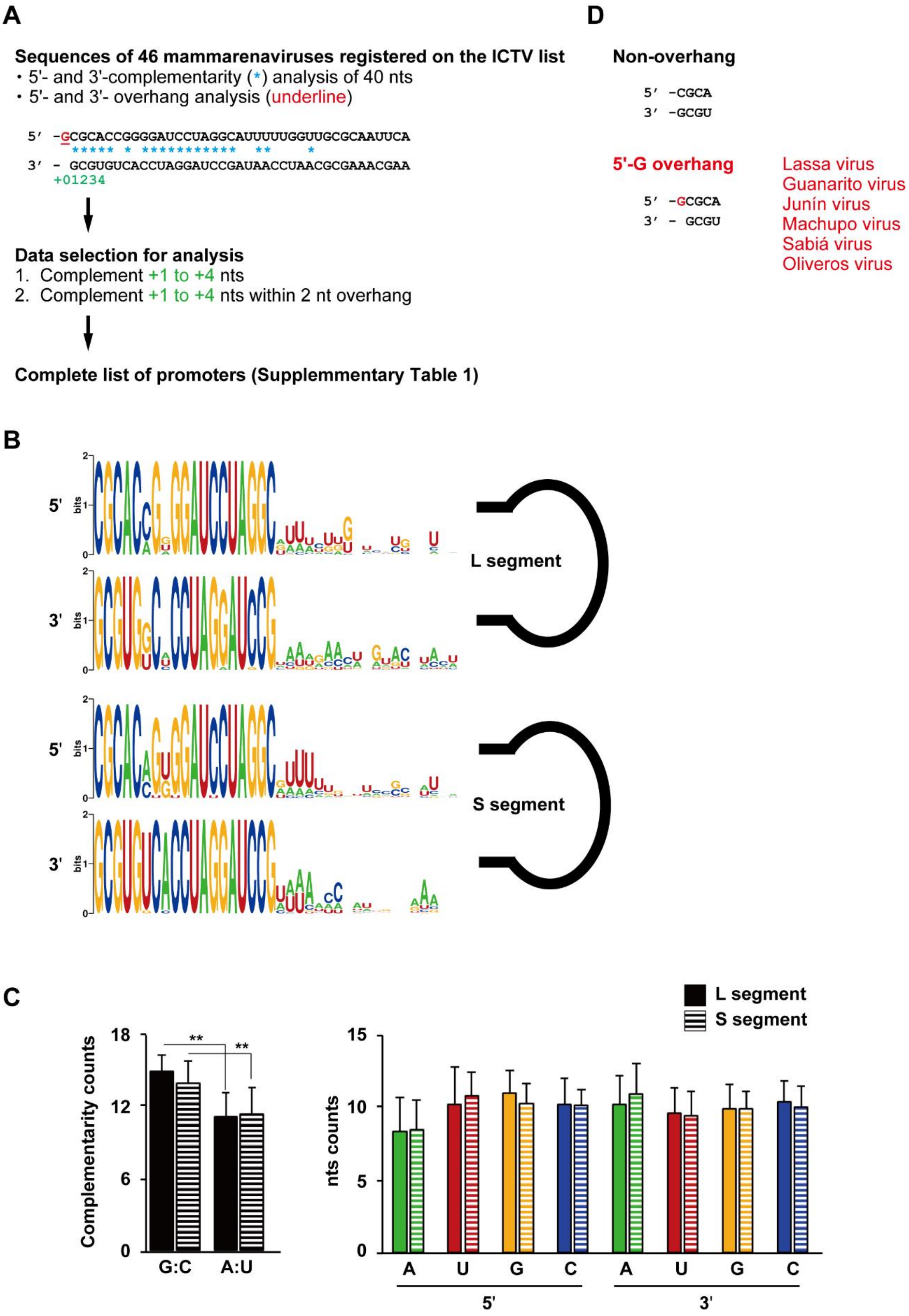
A
Sequences of 46 mammarenaviruses registered on the ICTV list
• 5'- and 3'-complementarity (*) analysis of 40 nts
• 5'- and 3'- overhang analysis (underline)
5′ -GCGCACCGGGGAUCCUAGGCAUUUUUGGUUGCGCAAUUCA
***** * *********** ** *
3′ - GCGUGUCACCUAGGAUCCGAUAACCUAACGCGAAACGAA
+01234
Data selection for analysis
1. Complement +1 to +4 nts
2. Complement +1 to +4 nts within 2 nt overhang
Complete list of promoters (Supplemmentary Table 1)
D
Non-overhang
5′ -CGCA
3′ -GCGU
5'-G overhang
5′ -GCGCA
3′ - GCGU
Lassa virus
Guanarito virus
Junín virus
Machupo virus
Sabiá virus
Oliveros virus
B
5'
3'
bits
L segment
S segment
C
L segment
S segment
Complementarity counts
18
12
6
0
G:C
A:U
**
**
nts counts
15
10
5
0
A
U
G
C
5'
3'

Supplementary Figure 1. Construction of the list of promoters in *Arenaviridae*

(A) The genome of the family *Arenaviridae* possesses an unpaired nt at the genomic end that forms an overhang. To account for this overhang in the analysis, sequences that showed complementarity between +1 and +4 within 0- to 2-nt shifts in the 5' or 3' ends of all arenavirus genomes were selected. In total, 25 arenavirus promoter sequences, as listed in Supplementary Table 2, were included (2 tri-segmented antennaviruses, and 23 di-segmented mammarenaviruses). (B) The conservation of the extreme 38 nts (without the overhang) in each of the 5' and 3' ends of the L and S segments among the 23 mammarenavirus species was analyzed. (C) The counts of G:C and A:U complementarity, and A, U, G, and C in the first 40 nts (for 38 to 39 nts in the opposite strand of 2-nt and 1-nt overhangs, respectively) of the L and S segments were determined. G:C complementarity was significantly higher than A:U complementarity in both segments (**p < 0.01, one-way ANOVA followed by Tukey's test), unlike in tri-segmented bunyaviruses. (D) A limited number of mammarenavirus species possessed an overhang nt in the database. It should be noted that many sequences of *Arenaviridae* genomes annotated in the NCBI database did not have overhanging genomic ends.

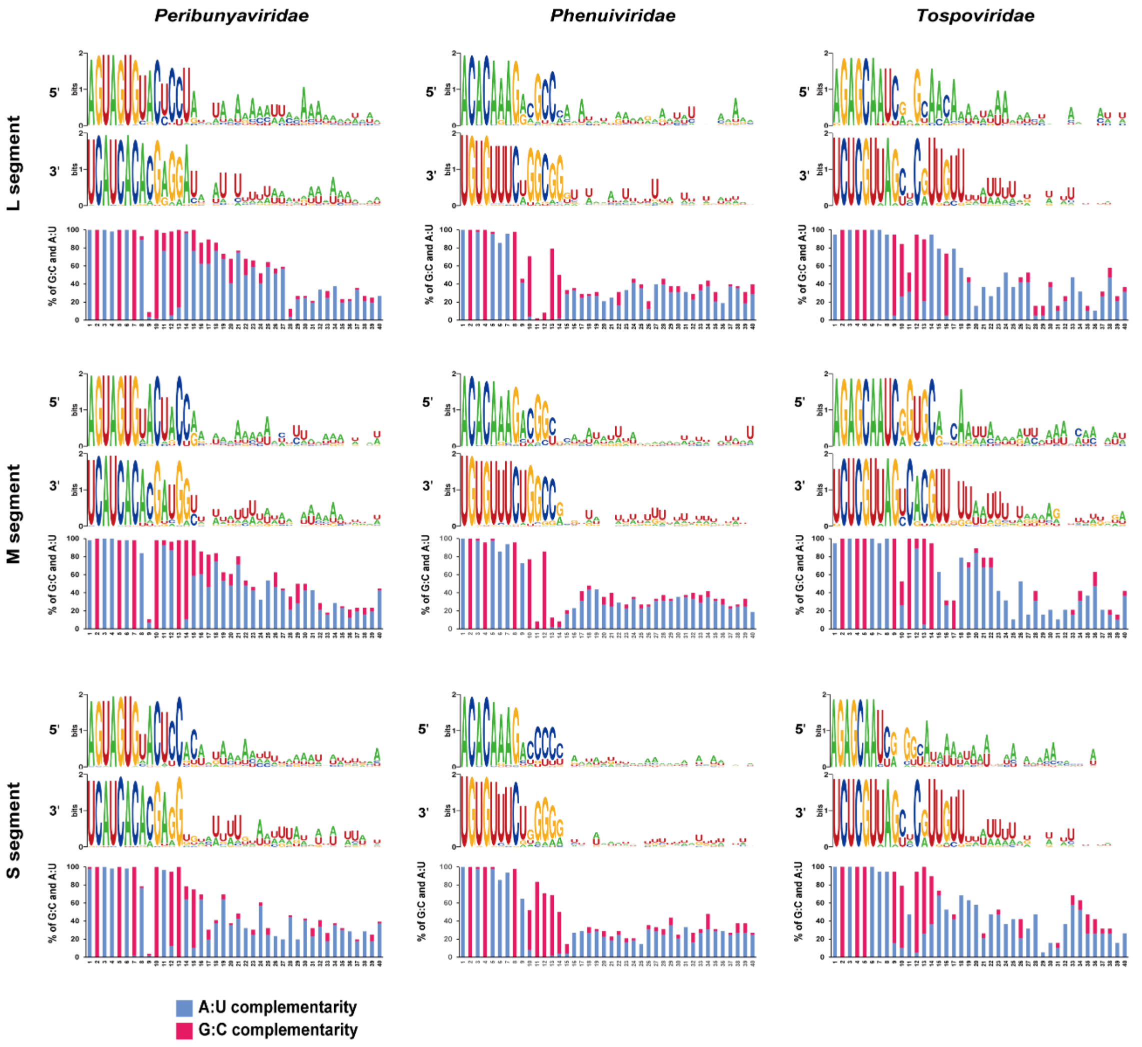

Supplementary Figure 2. Characteristics of the replication promoters of the L, M, and S segments of *Peribunyaviridae*, *Phenuiviridae*, and *Tospoviridae*

Sequence conservation was analyzed using the sequence logo generator WebLogo. The percentages of G:C and A:U complementarity in the promoter region (1 to 40 nts) among virus species in each family are shown as a bar graph. Virus species in the following families were examined: *Peribunyaviridae* (n = 56), *Phenuiviridae* (n = 47), and *Tospoviridae* (n = 19).

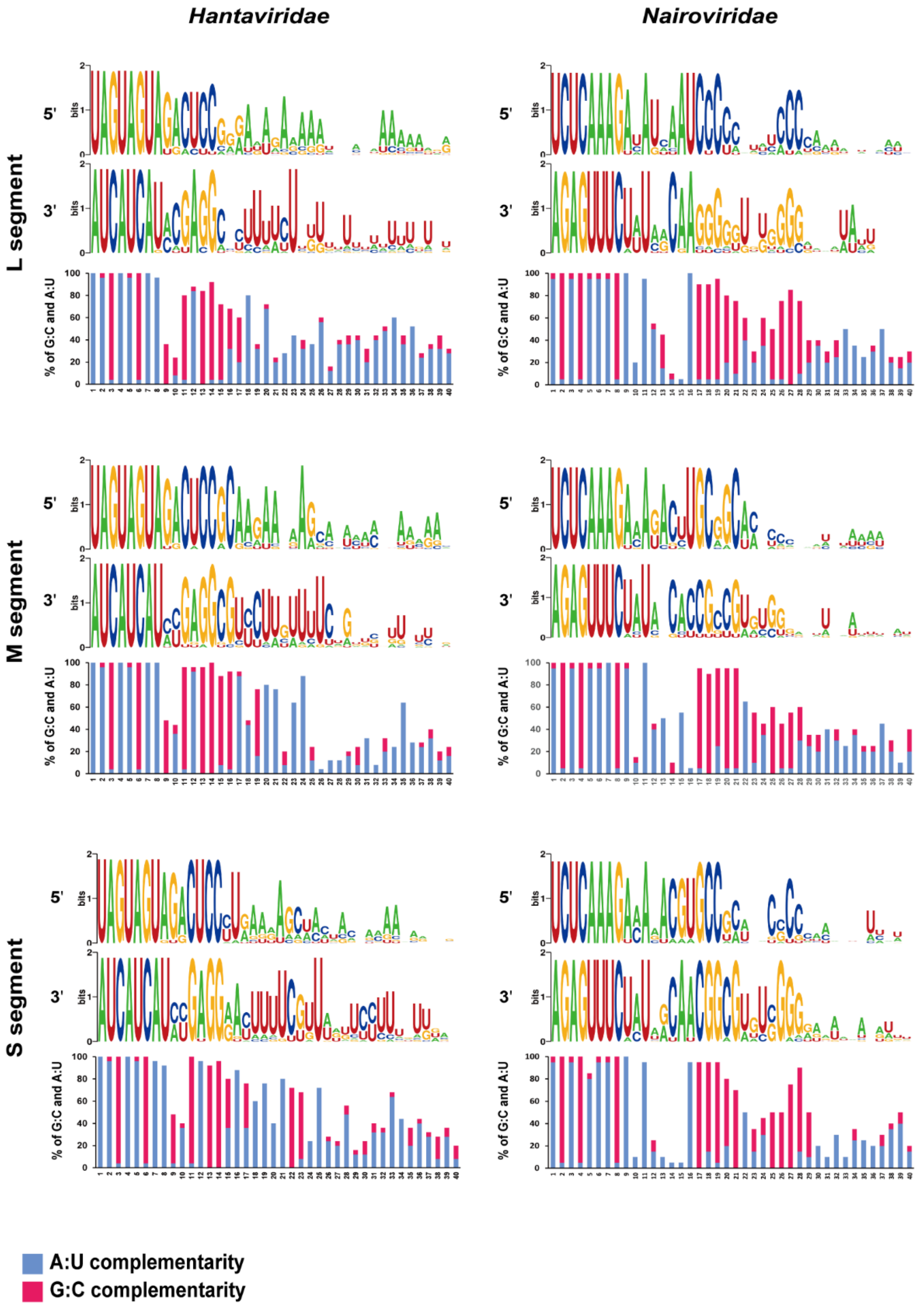

Supplementary Figure 3. Characteristics of the replication promoters of the L, M, and S segments of *Hantaviridae* and *Nairoviridae*

Sequence conservation was analyzed using the sequence logo generator Weblogo. The percentages of G:C and A:U complementarity in the promoter region (1 to 40 nts) among virus species in each family are shown as a bar graph. Virus species in the following families were examined: *Hantaviridae* (n = 24) and *Nairoviridae* (n = 20).

Supplementary Table 1. The automatic promotor calculator

Supplementary Table 2. Promoter list

Supplementary Table 3. *Nairoviridae* genome length
